# Self-Related Stimuli Decoding With Auditory and Visual Modalities Using Stereo-Electroencephalography

**DOI:** 10.3389/fnins.2021.653965

**Published:** 2021-05-04

**Authors:** Huanpeng Ye, Zhen Fan, Guohong Chai, Guangye Li, Zixuan Wei, Jie Hu, Xinjun Sheng, Liang Chen, Xiangyang Zhu

**Affiliations:** ^1^State Key Laboratory of Mechanical System and Vibration, School of Mechanical Engineering, Shanghai Jiao Tong University, Shanghai, China; ^2^Department of Neurosurgery of Huashan Hospital, Fudan University, Shanghai, China

**Keywords:** stereo-electroencephalography, response classification, auditory/visual modality, name decoding, self-referencing

## Abstract

Name recognition plays important role in self-related cognitive processes and also contributes to a variety of clinical applications, such as autism spectrum disorder diagnosis and consciousness disorder analysis. However, most previous name-related studies usually adopted noninvasive EEG or fMRI recordings, which were limited by low spatial resolution and temporal resolution, respectively, and thus millisecond-level response latencies in precise brain regions could not be measured using these noninvasive recordings. By invasive stereo-electroencephalography (SEEG) recordings that have high resolution in both the spatial and temporal domain, the current study distinguished the neural response to one's own name or a stranger's name, and explored common active brain regions in both auditory and visual modalities. The neural activities were classified using spatiotemporal features of high-gamma, beta, and alpha band. Results showed that different names could be decoded using multi-region SEEG signals, and the best classification performance was achieved at high gamma (60–145 Hz) band. In this case, auditory and visual modality-based name classification accuracies were 84.5 ± 8.3 and 79.9 ± 4.6%, respectively. Additionally, some single regions such as the supramarginal gyrus, middle temporal gyrus, and insula could also achieve remarkable accuracies for both modalities, supporting their roles in the processing of self-related information. The average latency of the difference between the two responses in these precise regions was 354 ± 63 and 285 ± 59 ms in the auditory and visual modality, respectively. This study suggested that name recognition was attributed to a distributed brain network, and the subsets with decoding capabilities might be potential implanted regions for awareness detection and cognition evaluation.

## 1. Introduction

One's name is one of the most socially self-related stimuli. According to the “cocktail party phenomenon,” a quiet whispering of one's name can arouse awareness even in a noisy environment (Wood and Cowan, 1995; Getzmann et al., 2017). Because of the specific emotional contents, a person's name has the preferential status (Tacikowski et al., 2011; Blume et al., 2017). Cognitive neuroscientists have highlighted the differences between the brain responses to one's own name and other's name. For instance, studies based on fMRI have demonstrated that stimulus of own name elicited unique brain functional activations in the medial prefrontal cortex, inferior frontal gyri, anterior cingulate, and anterior insula, while these activations were not shown under the stimulus of other's name (Carmody and Lewis, 2006; Tacikowski et al., 2011, 2013; Qin et al., 2012). Also, in electroencephalography (EEG) studies, the own name could elicit unique event-related potentials (ERP) (Tateuchi et al., 2012; Tamura et al., 2016). In other cases, the own name resulted in higher P300 (a positive-going ERP) amplitude and shorter latency compared to other names (Tacikowski and Nowicka, 2010; Kotlewska and Nowicka, 2015).

The preferential status of own name was different between healthy subjects and patients with cognitive disorders, thus for clinical practice, the name presentation paradigm has the potential to assess cognition and detect awareness, which serves as a cognitive brain-computer interface (BCI). Among adults and infants, own name elicited enhanced P300 when compared to other's names in the healthy control group, whereas individuals with autism spectrum disorder (ASD) showed similar P300 responses to the two names (Parise et al., 2010; Cygan et al., 2014; Arslan et al., 2020), and also indicated a disrupted or altered task-related connectivity (Nowicka et al., 2016). Additionally, for patients with disorders of consciousness (DOC, e.g., chronic coma), cognitive ERPs were also highly associated with awakening. Thus, responses of names could be an index to estimate the degree of conscious awareness. There has been evidence that the response to own name, contrasted to a response to any other name, can serve as a tool for brain function assessment in prolonged DOC patients (Sergent et al., 2017; Kempny et al., 2018; Li et al., 2018b).

Most of the existing name-related studies adopted non-invasive recording techniques of physiological activities. Scalp-EEG recording has been widely applied in name recognition tasks (Pan et al., 2014; Nowicka et al., 2016). However, it is challenging to investigate precise brain regions associated with name processing by scalp-EEG recording, due to its superficial recording and low spatial resolution. FMRI provides a whole-brain scan with high spatial resolution, but the rapidly changing neural processing can not be captured by fMRI due to its low temporal resolution. High spatiotemporal resolution is essential to the measurement of millisecond-level response latencies in precise brain regions. In comparison, a minimally invasive approach, stereo-electroencephalography (SEEG), measures neural activities directly by inserting depth electrodes containing multiple recording contacts into the human brain, and thus has balanced millimeter-level spatial resolution and millisecond-level temporal resolution (Parvizi and Kastner, 2018). SEEG can record distributed cortical and subcortical structures simultaneously. Therefore, using abundant temporal and spatial information, SEEG has the potential to decode name responses, measure response latencies, and locate precise response regions over a large spatial scale. Furthermore, the contribution for classification of each precise region can be evaluated by SEEG, which can not be implemented using scalp-EEG or fMRI.

In this study, we aimed to identify effective features to distinguish name responses in precise regions. Therefore, we recorded the neural responses to the own name and a stranger's name from multiple SEEG subjects, where the two different names were presented to each subject both acoustically and visually. Then we evaluated the feasibility and performance of decoding these two types of cognitive responses via spatiotemporal features of SEEG. The results show that these two different names could be predicted using SEEG signals recorded from multiple brain regions, where the high-frequency activity produced superior decoding accuracy within the used spectral features. Besides, the brain regions, including the supramarginal gyrus (SMG), middle temporal gyrus (MTG), superior temporal gyrus (STG), and insula could provide rich neural information for the decoding process under both stimulus modalities.

## 2. Materials and Methods

### 2.1. Subjects

Nine right-handed subjects (Table 1) participated in this study. All subjects were intractable epilepsy patients undergoing SEEG monitoring for seizure localization. SEEG electrodes implanting configurations were determined strictly for diagnostic purposes rather than the needs of this study. All subjects signed informed consent, which was approved by the Ethics Committee of Huashan Hospital, Shanghai, China (No. 2019-518).

**Table 1:** Subject demographics, implanting information, neural recording details.

**Subject**	**Age** **(years)**	**Implanting** **information**	**Sampling** **frequency**	**Trial** **size per** **session**	**Cue and** **preparation** **existence**	**Stimulus** **duration** **(ms)**	**Inter-trial** **interval** **(ms)**
S1	30	Left (138), Right (32)	2,000	80	–	2,000	2,000
S2	31	Left (144)	2,000	60	–	2,000	2,000
S3	27	Left (114), Right (30)	2,000	80	–	2,000	2,000
S4	24	Right (104)	2,000	120	–	1,000	1,000
S5	24	Right (108)	1,000	120	–	1,000	1,000
S6	16	Left (104), Right (33)	2,000	120	*	1,000	1,100–1,300
S7	33	Left (150)	2,000	120	*	1,000	1,100–1,300
S8	15	Right (102)	2,000	120	*	1,000	1,100–1,300
S9	32	Left (30), Right (96)	2,000	120	*	1,000	1,100–1,300

The numbers in brackets in the column of “Implanting information” indicates the number of contacts in the hemisphere. The “*” or “−” in the column of “Cue and preparation existence” indicates whether or not the subject received the cue and preparation time before the name presentation.

### 2.2. Experimental Paradigm

**Figure 1:**
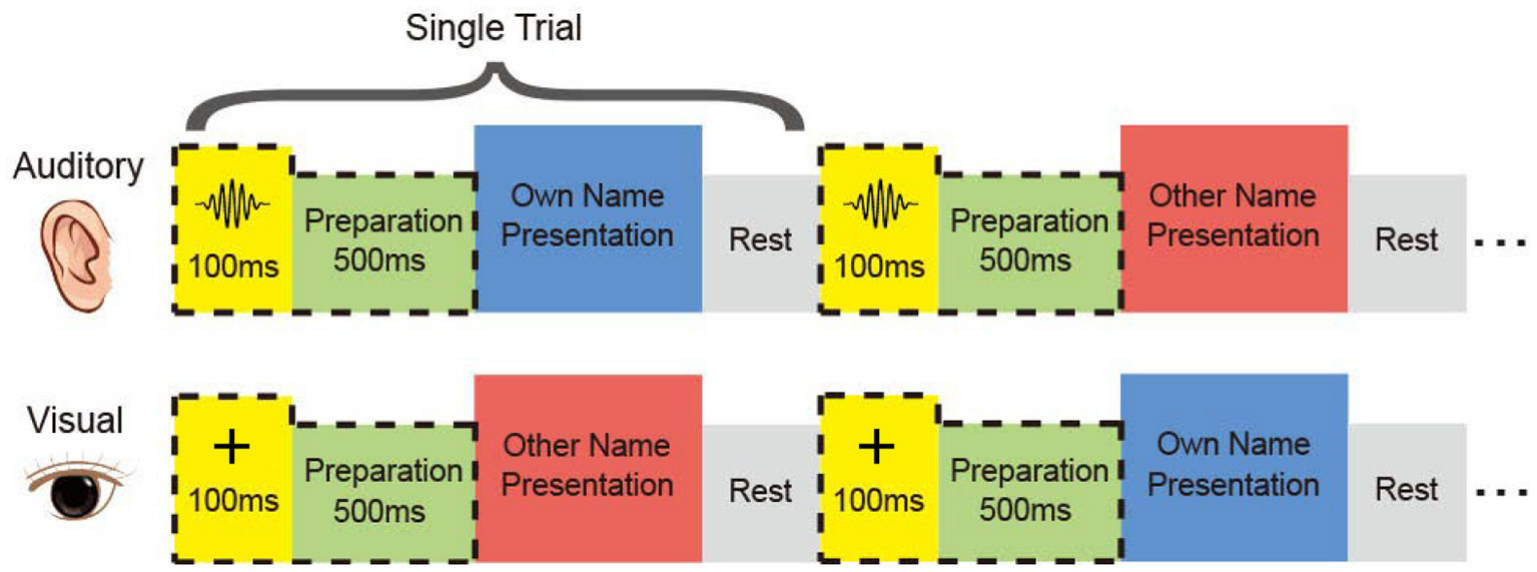
Experimental paradigm. In each session, the subject received his name and the other name aurally or visually. The two names were repeated in a pseudo-random sequence. Subjects performed mental recognition of the name. Four of the subjects heard a short burst in the auditory session and saw a cross in the visual session before the name presentation.

It was possible that differences between responses of the two names were not caused by the inner meanings of the names, but different pronunciations and intonations in the auditory modality, or glyphs in the visual modality. But for regions showing cross-modal high accuracies, it was more likely that the difference represented the inner meanings of the names. To investigate the distribution of cross-modal regions, we designed the name-presented experiment, which contained the auditory session and the visual session (Figure 1), and the two sessions were implemented successively. All subjects participated in both sessions and received their customized stimuli, namely the subject's full name and a stranger's full name. The two names had the same length, but with different pronunciations. In the auditory session, the auditory stimulus was presented to each subject by an in-ear headphone. Two different names were repeated for fair trials in a pseudo-random sequence. In the visual session, the own name and the control (other) name were presented to the subject using a 23 inch LCD monitor.

The subject was asked to discriminate his/her name and the other name in the mind, without other reactions and movements during the experiment. For each subject, the two sessions consisted of the same number of trials with equal duration of the stimulus. Trial size, duration of the stimulus, and inter-trial interval kept unchanged across the auditory and visual tasks for each subject, shown in Table 1. After preliminary analysis for the first three subjects S1, S2, and S3, we found that 1 s stimulus duration was enough to elicit the subject's response, and 1 s inter-trial interval was enough for the response to revert to the baseline. Therefore, the stimulus duration and inter-trial interval were set as 1 s for the following subjects. Furthermore, to avoid the subject's adaption, another floating inter-trial interval from 100 to 300 ms was added (Nowicka et al., 2016) for subjects S6, S7, S8, and S9, and these subjects received a 100 ms cue and 500 ms preparation time before the name presentation to attract attention. The cues were a short burst of sound in the auditory session and a fixation cross in the visual session, respectively.

### 2.3. Data Recording and Electrode Localization

**Figure 2:**
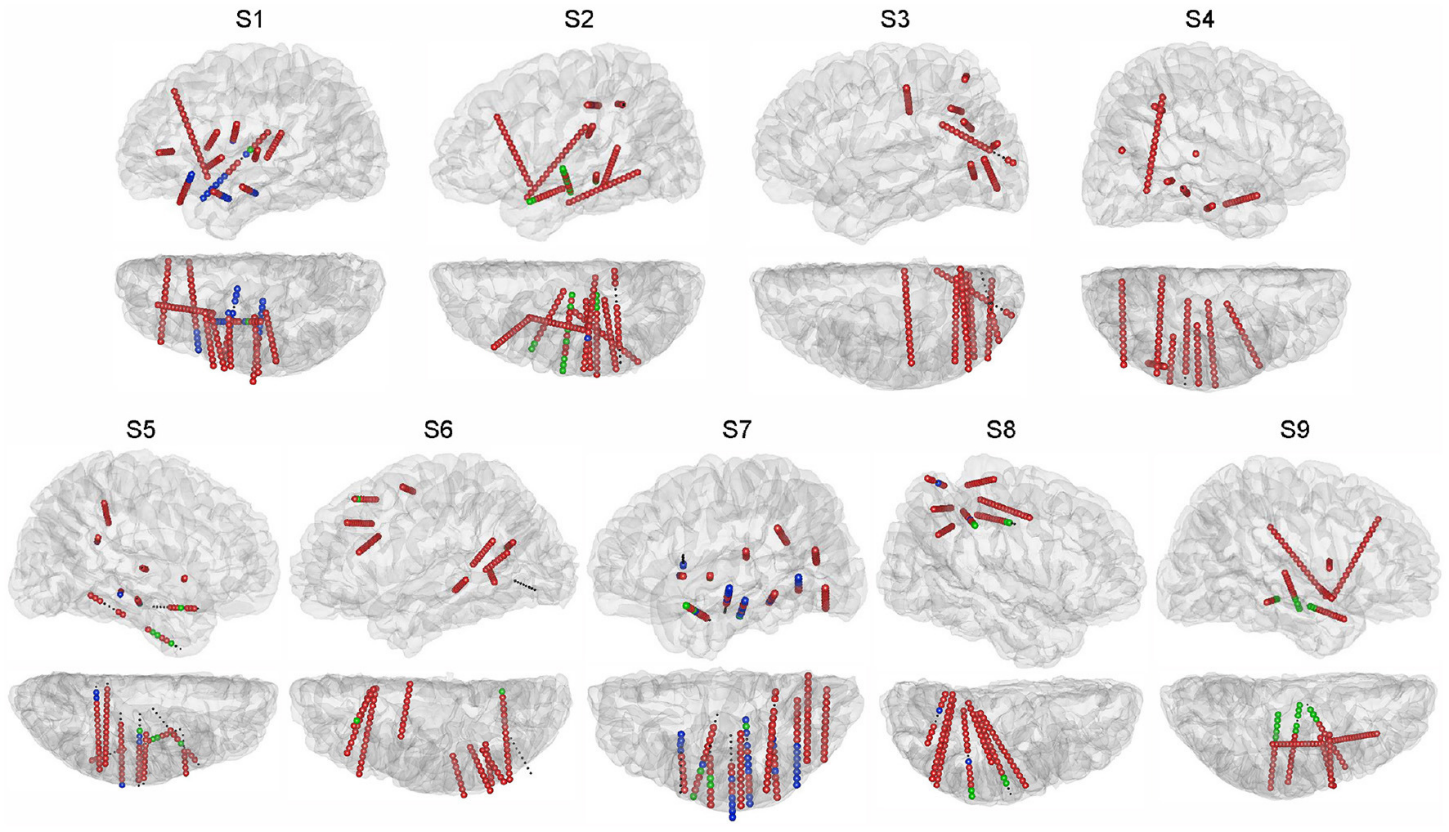
Electrode locations projected on the individual brain. For each subject, sagittal and transverse views of the hemisphere with electrodes are shown. Subjects S1, S3, S6, and S9 have electrodes in both hemispheres, and only the hemispheres containing the majority of electrodes are shown. Different colors of the SEEG contacts indicate the session in which the contact was utilized: green for the auditory session, blue for the visual session, red for both sessions. Small black dots indicate the SEEG contacts excluded from this study.

SEEG data were recorded with a clinical recording system (EEG-1200C, Nihon Kohden, Irvine, CA) and were digitized at 1,000 or 2,000 Hz. Each depth electrode shaft contains 8-16 contacts. Each contact is 2 mm long with a 0.8 mm diameter and 1.5 mm spacing distance. For each subject, using pre-implant MRI and post-implant CT images, we first rebuilt the individual brain by performing brain reconstruction and segmentation in Freesurfer (Fischl et al., 2002), which parcelled a range of different cortical areas and also subcortical structures. Based on results from Freesurfer, we then identified the 3D coordinates and the anatomical labels within the brain for all the SEEG contacts with the iEEGview Matlab toolbox (Li et al., 2019) (Figure 2).

### 2.4. Signal Pre-processing

Neural activities from frequency bands, high-gamma (60–145 Hz), beta (13–30 Hz) (Kirkby et al., 2018; Sani et al., 2018), and alpha (8–12 Hz) were examined, and the corresponding spatiotemporal features were extracted based on their band power, respectively. Offline data processing was implemented in the Matlab platform. First, the contacts whose line noise power at 50 Hz was larger than a significance level were removed from further analysis. The significance level was defined as median line noise power across all contacts plus ten times their median absolute deviation. Then a 50 Hz comb notch filter was applied to remove the possible line noises and their harmonics. Second, we referenced the signals using the Laplacian reference method (Li et al., 2018a). In brief, the signals of each time point were subtracted by the average signals from adjacent contacts at the same time point. Third, we band-pass filtered the signals using a 6th-order Butterworth filter at high-gamma, beta, and alpha frequency bands separately. The power trace of each contact at each frequency band was extracted by squaring the absolute value of the Hilbert transformed signals. The derived power trace was divided into trials according to the markers at the stimulus onset. For each trial, we defined the 200 ms interval preceding the stimulus onset as the baseline, and normalized the power trace from 0 to 1,000 ms following the stimulus onset using Z-scored transformation against the baseline. This 1,000 ms power trace under name presentation was used for the feature extraction. The trace of each trial was convoluted with an 80-ms Gaussian window for smoothing (Miller et al., 2016). We computed the mean and standard error of the power trace across trials for each contact, and the contacts influenced by artifacts were removed, where the standard error of power across trials was larger than its average at a time point. The pre-processing procedure kept 1,027 (auditory session) and 1,041 (visual session) contacts out of 1,185 contacts in total (Figure 2).

### 2.5. Feature Extraction and Dimensional Reduction

The procedure of feature extraction was performed for each subject at each frequency band separately, with the purpose of identifying the suitable frequency band to classify the two names. For each contact, the 1,000 ms power trace following the stimulus onset was separated into 100 non-overlapping bins with a length of 10 ms. We calculated the mean power amplitude in each bin and concatenated them as *v*. The feature vector of each trial, *V*, was generated by splicing *v* from all contacts. Considering there might be redundancy in such a high dimensional feature vector *V*, according to the training set, we then implement a permutation test to select the most informative features for decoding at each frequency band (Schalk et al., 2007; Adriana et al., 2016; Li et al., 2018a). The permutation test was performed for each feature dimension. In the first step of the permutation test, for each feature, we aligned its values across all own/other name trials as *x* and *y*, separately, and then concatenated *x* and *y* as *z* and then correlated *z* with the corresponding labels to obtain the Spearman *r*-value. In the second step of the permutation test, we randomly shuffled the own/other labels and calculated the *r*-value between *z* and randomized labels. Then the randomization step was repeated 1,000 times, and thus, generating a Gaussian distribution with 1,000 surrogate *r*-values. The *p*-value was analyzed, which was the percentage of the original *r*-value belonging to the Gaussian distribution. The significance level was set as 0.05 divided by the feature dimension of *V* according to Bonferroni correction. Finally, *p*-values of all features were calculated, and the 20 most informative features with the smallest *p*-value were extracted. The selected 20 features were derived from different contacts and time bins among subjects. For a subject, the 20 features came from 1 to 10 contacts, and they were all significant. The 20-dimensional feature vector (*V*_20_), representing task-related spatial and temporal information, was used for the following classification process.

### 2.6. Classification

#### 2.6.1. Classifier

The classification was implemented within each frequency band. Linear discriminant analysis (LDA) and random forest were used separately to verify the consistency of the analysis results. For the LDA classification, the extracted feature set *V*_20_ was first subjected to a principal component analysis, and the number of principal components used for the classification corresponded to >90% of explained variance, which was 2–6 in different subjects. For the random forest, 200 decision trees were chosen to avoid overfitting (Liaw and Wiener, 2002), where each tree randomly selected a subset of *V*_20_ and generated a decision independently, and then votes of all trees generated the final decision of the random forest (Breiman, 2001).

#### 2.6.2. Validation

**Figure 3:**
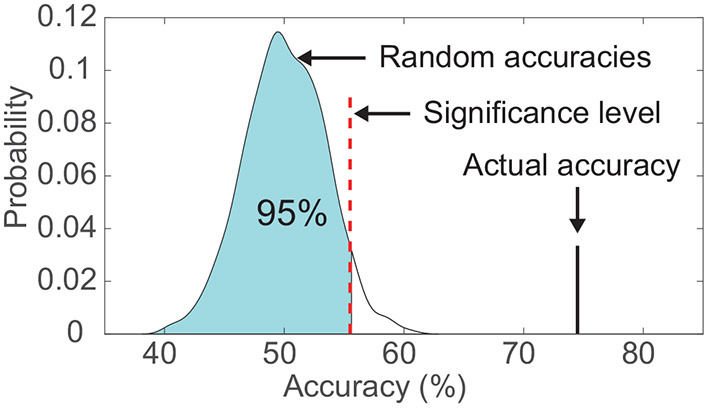
Comparison between the actual classification accuracy and the empirical distribution of random accuracies. The significance level was set as the accuracy corresponding to the 95th percentile of the empirical distribution. This example data was from subject S6's visual modality.

For both classifiers, we evaluated the classification using a leave-one-out cross-validation (Sani et al., 2018) within each subject. In brief, a single-trial *V*_20_ was left out to be predicted, and the rest of trials were used for fitting the decoding model, therefore, there was no overlapping between the training trials and the test trial. Additionally, the statistical significance of the classification result was calculated using a permutation test, which aimed to verify that the classification accuracy exceeded the chance level significantly (Figure 3). In detail, for each subject, we kept the output labels of the classifier unchanged, and randomly shuffled the actual labels across classes. Then the permuted classification accuracy was calculated using pairs between the unchanged output labels and the permuted actual labels. This procedure was repeated 1,000 times under the same frequency band and classifier, and the significance level of classification accuracy corresponded to the 95th percentile (*p* < 0.05) of the empirical distribution established by randomly permuting the data (Figure 3; Combrisson and Jerbi, 2015; Branco et al., 2016). A two-way ANOVA was performed to test the significant difference in the performance of the three frequency bands (high-gamma, beta, and alpha) and the two classifiers (LDA and random forest).

#### 2.6.3. Contribution to Classification

All contacts were clustered according to their anatomic locations for each subject. To further explore the brain regions' contribution to the classification accuracy, for each subject, we first identified the region that contributed the most features to *V*_20_, when multi-region SEEG contacts were used. Second, classification accuracies of single regions were calculated. In brief, for each region within the subject, we implemented the same feature extraction procedure again using contacts in the single region, and then calculated the classification accuracy of this single region.

### 2.7. Identification of Cross-Modal Regions

We then addressed whether each subject had specific single regions, that both their auditory and visual classification accuracies exceeded the significance levels (section 2.6.2), and we termed them as cross-modal regions. For each modality, to quantify the extent to which the classification accuracy was caused by the physical difference between the two stimuli, we evaluated the physical difference between stimuli using cross-correlation, and then implemented a least-square linear regression analysis between the cross-correlation values and the classification accuracies in the cross-modal regions. Besides, the response latency in these cross-modal regions was calculated. In each contact, we identified the first time point in which the response to own/other name showed significant difference compared with the baseline (the rest state), and identified the first time point with a significant difference between responses to own name and other name.

#### 2.7.1. Physical Difference Between Stimuli

Naturally, the two names presented by the in-ear headphone in the auditory modality had different instantaneous amplitudes, which was the most distinctive characteristic to distinguish the two sound sequences in our case. Therefore, the difference between the two names was assessed by the cross-correlation of their instantaneous amplitude. In detail, we first extracted the amplitude envelopes of the two sound sequences using the Hilbert transform, and then evaluated the difference of the two envelopes by the Matlab function *xcorr*, which returned the cross-correlation of the two time sequences. We used the cross-correlation value corresponding to 0 lag between the two sequences. In the visual modality, we adopted an approach in image processing to compare the two names presented by the monitor. In detail, the difference between the two name images was evaluated by the Matlab function *xcorr2*, which was the two-dimensional version of *xcorr*. We used the cross-correlation value corresponding to 0 shift between the two images. Finally, the cross-correlation values were normalized in the range from −1 to 1.

#### 2.7.2. Response Latency

For each contact in the cross-modal region, the response latency of own/other name was first calculated using a permutation test similar to that in section 2.5. In detail, we first got the average high-gamma power amplitude in the 200 ms baseline (rest state) for each trial, and aligned its samples across all own name trials as *x*. At a time point (a bin with a length of 10 ms) after the stimulus onset, the amplitudes of all own name trials were aligned as *y*. Second, the permutation test was used to verify whether there was a significant difference between *x* and *y*. We explored the first time point that showed a significant difference between *x* and *y*, and termed it as the response latency of own name. The response latency of other name was calculated with the same procedure. Besides, using the same permutation test, we also explored the first time point in which the responses to own name and other name showed significant difference.

Besides the latency of response difference, the generation times of features in *V*_20_ were also analyzed. Further, the generation times of features in *V*_20_ depicted not only the first time point with a significant difference, but also all points with significant differences through the name presentation, which were used in the classification.

### 2.8. Controlling for Rest State

To further validate the proposed classification scheme and demonstrate the extent of the neural response under name presentation, we added a control scenario, where the baseline activity (rest state) was considered and a three-class classification was conducted. This three-class classification could further validate not only the classification between two name responses, but also the activations compared with the baseline. The 1,000 ms interval preceding the stimulus onset was used as the sample of the rest state, and then it was normalized by Z-scored transformation. Using multi-region contacts, we implemented the feature extraction again. Especially, the permutation test was conducted three times for dimensional reduction, because the spatiotemporal features were selected using three pairwise combinations among three classes (rest, own, and other). Each execution of the permutation test selected ten features, and thus the total feature dimension after permutation tests was thirty. The significance level was corrected by Bonferroni correction, and these thirty features were all significant. For each class, its sensitivity and precision were defined as follows:

(1) $$\begin{array}{r} Sensitivity=\frac{\text{TP}}{\text{TP}+\text{FN}}\times 100\% \end{array}$$

(2) $$\begin{array}{r} Precision=\frac{\text{TP}}{\text{TP}+\text{FP}}\times 100\% \end{array}$$

For each class, TP was the number of samples of the current class that identified by the classifier; FN was the number of samples of the class, which were missed by the classifier; FP was defined as the number of other-class samples, which were identified as the current class by the classifier. The definition of classification accuracy for each class was the same as the sensitivity. Therefore, the classification performance of each class was further assessed in terms of sensitivity and precision, confusion matrix, and receiver operating characteristic curve (ROC).

## 3. Results

### 3.1. Classification Performance of Multi-Region Contacts

**Figure 4:**
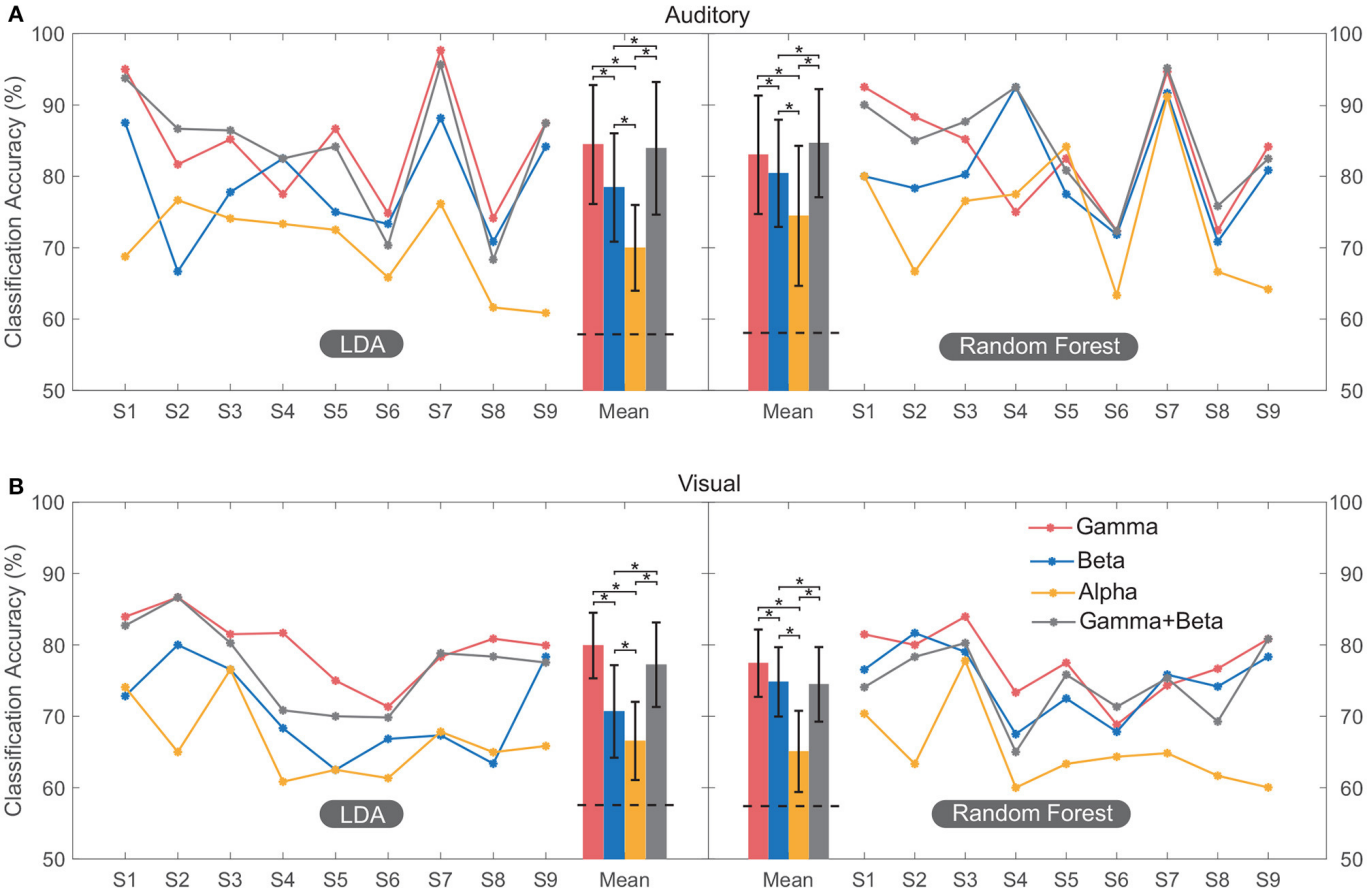
Classification performance of multi-region contacts for all the subjects. The accuracy of each subject was plotted by line graph, and their mean value was plotted by bar graph for convenient observation. In both auditory **(A)** and visual **(B)** modalities, classification was conducted using LDA and random forest. Error bars depict the standard deviation of the classification accuracies across subjects. The “*” indicates the significant difference. The horizontal black dashed line on the bars of mean accuracy indicates the mean significance level of classification (section 2.6.2) using high-gamma band across subjects.

Figure 4 shows the classification accuracy computed using different spectral features across multiple regions. The average accuracy across all subjects calculated using high-gamma power (81.2 ± 7%) was significantly higher (*F* = 18.89, *p* < 0.001) than that calculated using beta (76.1 ± 7.4%) and alpha power (69 ± 7.6%) based on all sessions. Moreover, the performance achieved using the combination of high-gamma power and beta power features (80.1 ± 8.2%) had no significant (*p* = 0.53) difference with that of high-gamma power only (Figure 4). Therefore, these results indicated that high-gamma power activity can provide the most distinguishable neural information for the classification. Only features from the high-gamma band were used in the following classification.

The average classification accuracies of the auditory session were 84.5 ± 8.3% (LDA) and 83.1 ± 8.2% (random forest). The average accuracies for the visual session were 79.9 ± 4.6% (LDA) and 77.7 ± 4.7% (random forest). Each of these classification accuracies exceeded its significance level (*p* < 0.05, section 2.6.2). There was no significant difference (*F* = 0.09, *p* = 0.75) between accuracies calculated using LDA and random forest, and thus only accuracies calculated using LDA were shown in the following classification.

### 3.2. Brain Regions Relevant to Classification

**Figure 5:**
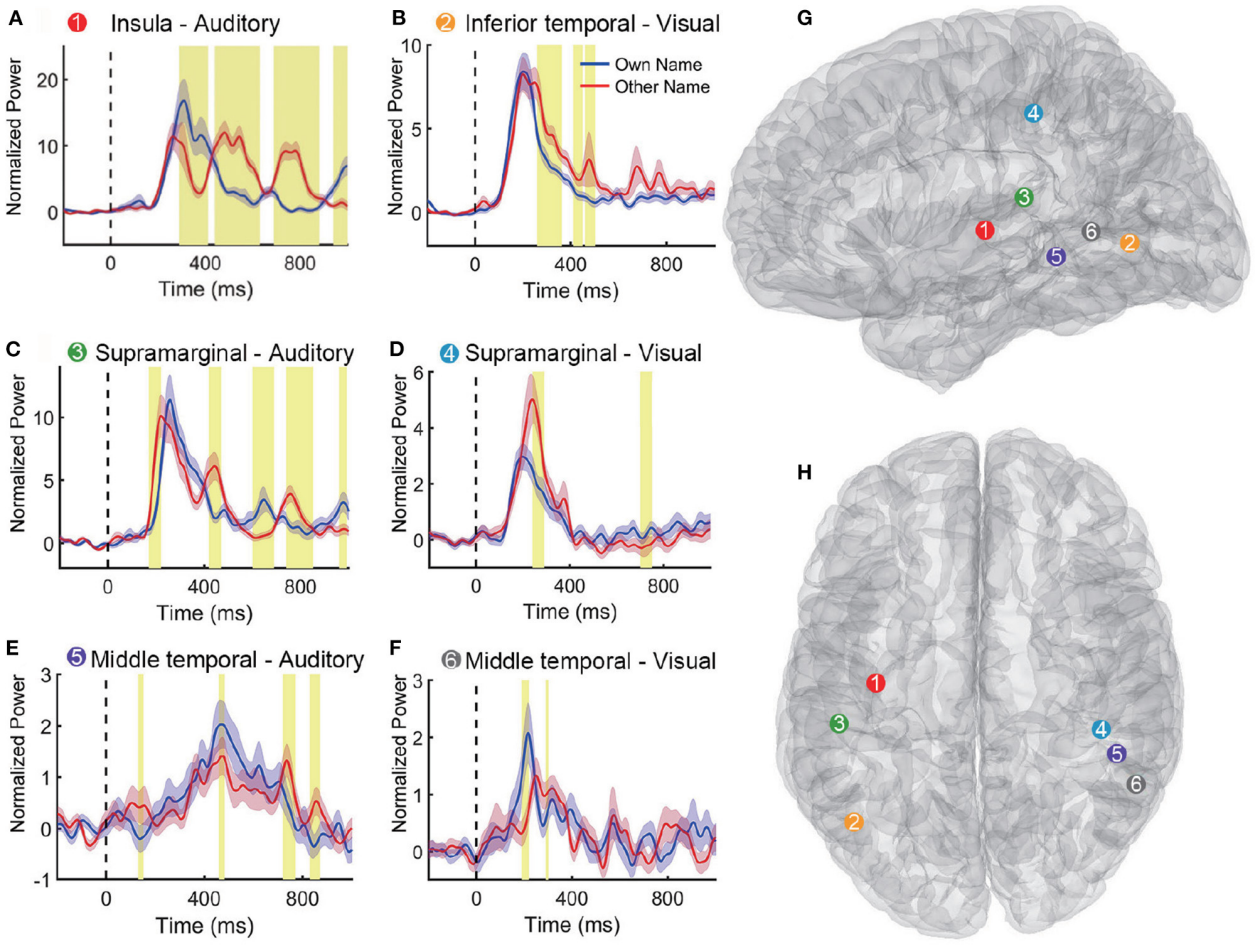
Representative high-gamma-power traces following own and other name presentation. **(A–F)** Shows responses to the two names in different contacts. The blue and pink shaded region represents the standard error across trials. The intervals highlighted with yellow indicate that there were significant differences between the two responses (*p* < 0.05). Vertical dashed lines (Time = 0 ms) indicate the stimulus onset. **(G,H)** Show six contact locations in the sagittal and transverse view of the standard MNI brain model. The response traces in **(A–F)** and the corresponding contact locations in **(G,H)** are matched with different numbers and colors.

Table 2 shows the classification accuracy using the single region, which contributed the most features to the feature set *V*_20_ when multi-region SEEG contacts were used. Results showed that only nine out of eighteen regions in Table 2 achieved the highest accuracy among all regions for the subject. In certain regions, including the left insula and the transverse temporal region of subject S1, the left transverse temporal and the STG of S7, as well as the STG of S9, the single-region accuracy of the auditory modality exceeded or approached 90% (Supplementary Figure 1), which was close to the multi-region accuracy. The above regions produced the best performance among all the regions and provided the majority of the features, where contacts in these regions tended to present prominently different responses to the two stimuli (own name and other name, Figure 5A). The results of permutation test in section 2.5 were shown in representative contacts in Figures 5A–F, where intervals highlighted with yellow indicated that there were significant differences between the two responses (*p* < 0.05). The locations of these representative contacts were shown in Figures 5G,H. In Figure 5A, there are continuous intervals with significant differences (*p* < 0.05). Though other regions such as the left inferior temporal region of S7 (Table 2) provided maximum features, they showed relatively low classification accuracy. Contacts in these regions showed similar responses to different stimuli (Figure 5B), and they had relatively less or shorter intervals with significant differences than contacts in Figure 5A.

**Table 2:** Regions providing maximum features in each session.

**Subject**	**Session**	**Region**	**Number of feature**	**Accuracy (%)**	**Highest accuracy**
S1	Auditory	Left insula/Left transverse temporal	9/11	90/95.1	*
	visual	Left insula	12	74.1	–
S2	Auditory	Left superior parietal	5	78.3	–
	visual	Left supramarginal	6	81.6	–
S3	Auditory	Right precuneus	6	76.5	*
	visual	Left superior parietal	7	69.2	–
S4	Auditory	Right superior temporal	7	73.3	*
	visual	Right inferior parietal	5	70.9	–
S5	Auditory	Right superior temporal	14	82.5	*
	visual	Right supramarginal	9	70	–
S6	Auditory	Left pars opercularis	7	61.3	–
	visual	Right caudal middle frontal	14	64.8	–
S7	Auditory	Left transverse/superior temporal	10/9	97.7/97.7	*
	visual	Left inferior temporal	19	71.8	*
S8	Auditory	Right precentral	8	70.8	*
	visual	Right precentral	6	68.4	*
S9	Auditory	Right superior temporal	20	88.4	*
	visual	Right superior temporal	8	70	–

The “*” or “−” in the column “Highest accuracy” indicates whether or not the region achieved the highest accuracy among all single regions for the individual.

### 3.3. Performance of Cross-Modal Regions

#### 3.3.1. Classification Accuracy

**Figure 6:**
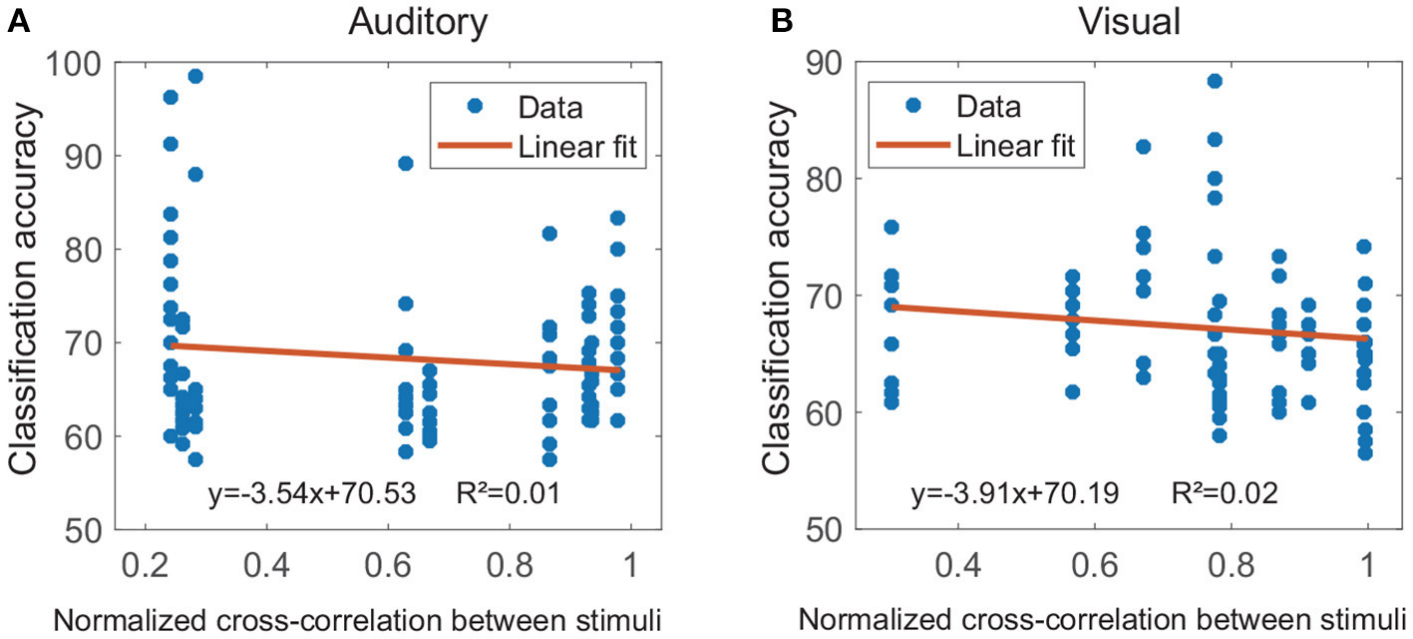
Results of the linear regression analyses between the physical difference of stimuli and the classification accuracy. The physical difference of stimuli was evaluated by the cross-correlation value, and several cross-modal regions of the same subject shared the same cross-correlation value for each modality. **(A,B)** Show results for the auditory modality and visual modality, respectively (*p* < 0.001).

The cross-modal regions were termed as single regions, that both their auditory and visual classification accuracies exceeded the significance levels. For easy to compare, in each subject, the significance levels of all single regions were uniformly set as the significance level resulted from multi-region contacts. The results showed that, for each subject, at least seven different cross-modal regions were found, and all cross-modal regions for each subject were shown in Supplementary Figure 1. Figure 6 shows the relationship between the physical difference of stimuli and the classification accuracies in cross-modal regions. During the calculation of linear regression analysis, several cross-modal regions of the same subject shared the same physical difference (cross-correlation value) for each modality. The result showed there was no correlation between these two measurements for both auditory modality (*k* = −3.54, *r*^2^ = 0.01, *p* < 0.001, Figure 6A) and visual modality (*k* = −3.91, *r*^2^ = 0.02, *p* < 0.001, Figure 6B), suggesting that the classification accuracy in the cross-modal region might be not caused by the physical difference between the two stimuli.

**Figure 7:**
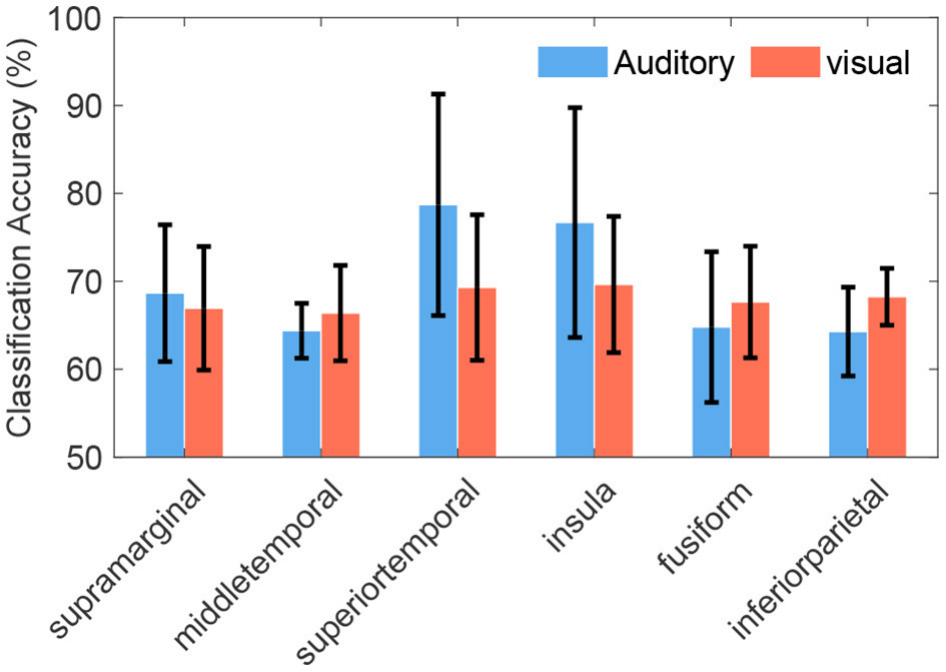
Average classification accuracies of cross-modal regions across subjects. These regions were common in at least 67% of subjects, even the supramarginal gyrus was common in all subjects. Error bars depict the standard deviation of the classification accuracies across subjects.

Some cross-modal regions were common across different subjects, such as SMG (in 100% of subjects), MTG (in 89% subjects), STG (in 78% subjects), insula, fusiform gyrus, and inferior parietal lobule (in 67% subjects). Representative cross-modal high-gamma responses of SMG were shown in Figures 5C,D, and cross-modal responses of MTG were shown in Figures 5E,F. Average classification accuracies of the above common cross-modal regions were shown in Figure 7. Among these common regions, STG achieved the highest auditory classification accuracy (78.9 ± 11.7%), and insula achieved the highest visual classification accuracy (69.8 ± 7.1%).

#### 3.3.2. Response Latency

**Figure 8:**
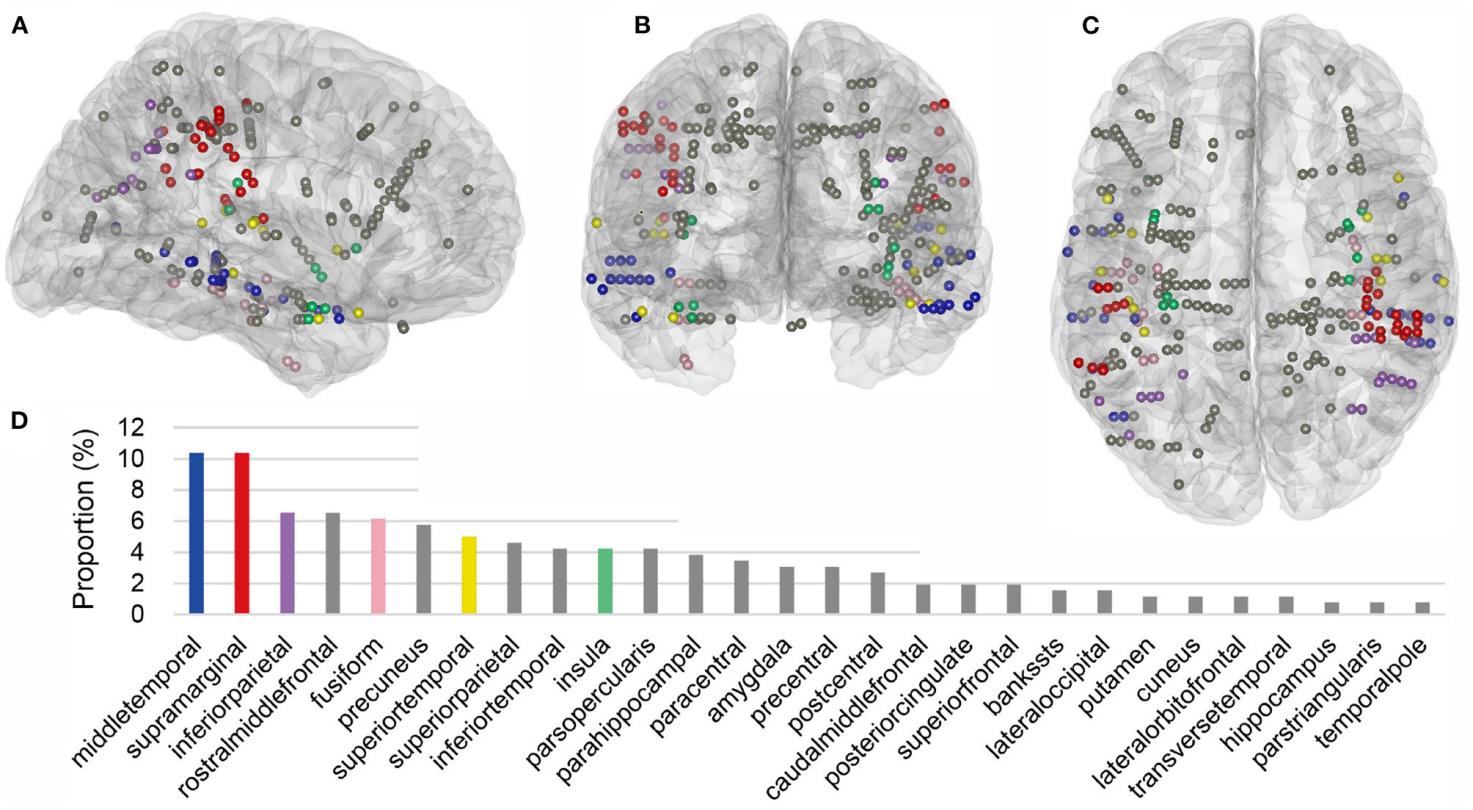
Cross-modal contacts projected on the three-dimensional standard Montreal Neurological Institute (MNI) brain model. **(A–C)** Show the brain model and cross-modal contacts in a sagittal, coronal, and transverse view, respectively. These contacts were all from cross-modal regions in Figure 7, and they provided informative features to both modalities. **(D)** Shows the proportion of single-region contacts in all cross-modal contacts. In brief, the number of total cross-modal contacts from all subjects was added, and the number of cross-modal contacts in each region from different subjects was divided by the total, generating the proportion. Common cross-modal regions in at least 67% of subjects were highlighted with different colors, and other regions were displayed with gray.

Within the cross-modal regions of each subject, we then further identified the locations of cross-modal contacts, which were defined as the contacts that contributed spatiotemporal features to *V*_20_ of the corresponding region in both auditory and visual modalities. Across all nine subjects, 260 cross-modal contacts were identified and the 3D locations of these cross-modal contacts projected into a standard Montreal Neurological Institute (MNI) brain model were shown in Figures 8A–C. Across the cross-modal regions, SMG and MTG had the most cross-modal contacts (27 in total for each region), accounting for 20.8% of the total (Figure 8D).

**Figure 9:**
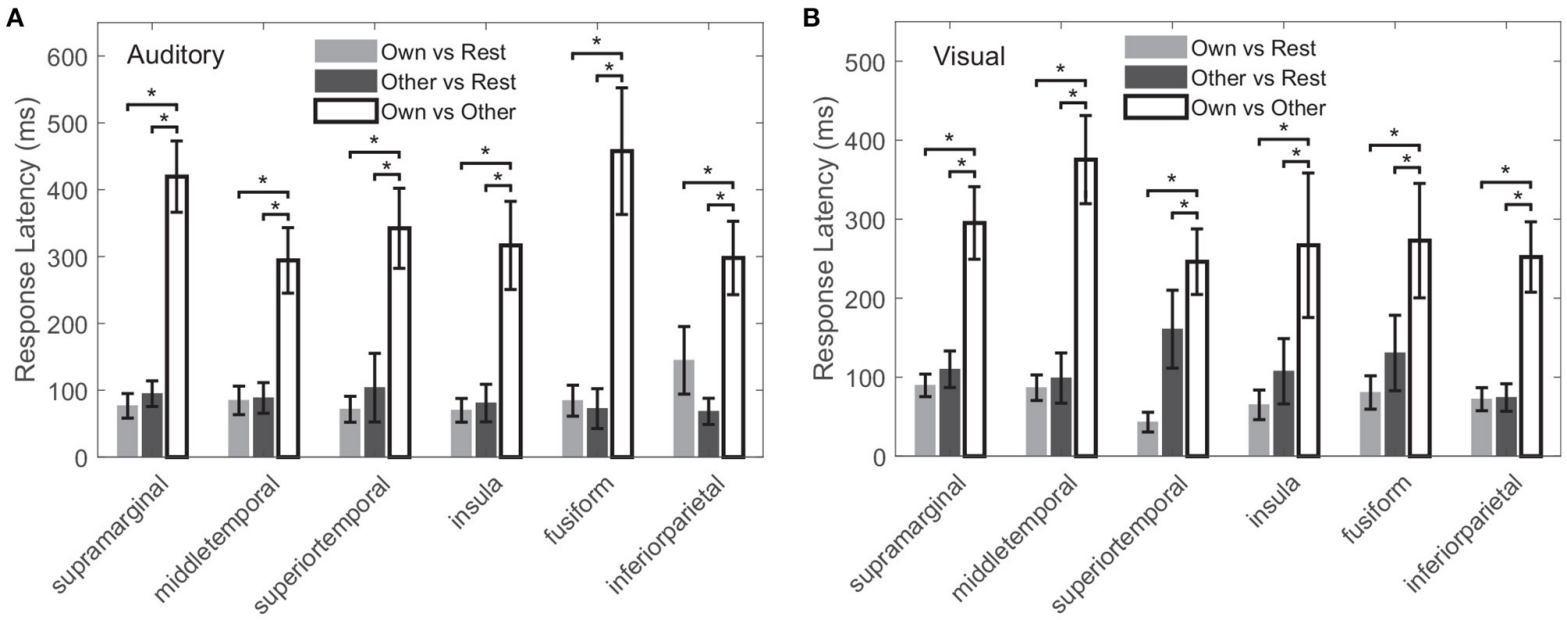
Latencies for own name activation, other name activation, and the difference between the two types of responses, marked as “Own vs. Rest,” “Other vs. Rest,” and “Own vs. Other,” respectively. **(A,B)** Correspond to the auditory and visual modality, respectively. The error bar indicates the standard error across all cross-modal contacts within the region. The “*” indicates the significant difference (*p* < 0.05).

The response latency in a cross-modal region was considered as the average latency across all cross-modal contacts within the region, shown in Figure 9. In all the above common cross-modal regions, compared to the baseline, latencies of the first activation for own name and other name were relatively short: in the auditory modality, the average latencies were 88 ± 25 and 85 ± 25 ms for own name and other name, respectively. Please note that the number following the “±” indicated the standard error across regions. In the visual modality, the average latencies were 72 ± 16 and 114 ± 35 ms for own name and other name, respectively. In contrast, the first time point in which own name and other name showed significant difference had a longer latency, and the values were 354 ± 63 and 285 ± 59 ms in the auditory and visual modality, respectively. In all common cross-modal regions, using cross-modal contacts from different subjects, a permutation test showed that there was no significant difference between latencies for own name activation and other name activation, while the latency for the own-other difference was significantly longer than the above two latencies (*p* < 0.05).

#### 3.3.3. Distribution of Generation Time of Features

**Figure 10:**
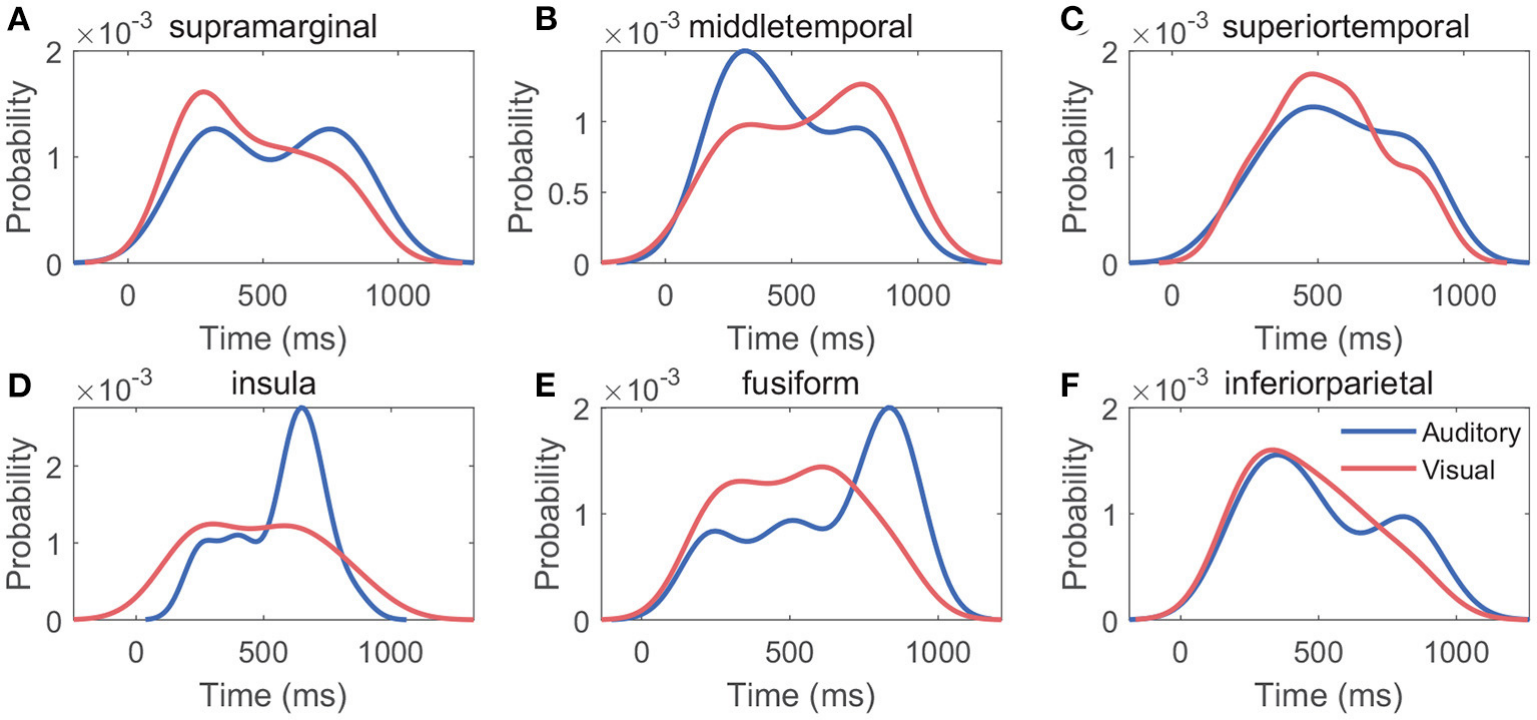
Distribution of generation time of features. **(A–F)** Indicate the single region's probability density function of generation time of features in *V*_20_. In each cross-modal region, the generation times of features derived all cross-modal contacts were used to generate the probability density function.

Figures 10A–F shows the probability density function of generation times of features in *V*_20_. For each cross-modal region, the generation time distribution of features varied between modalities, whereas the probability density functions reached a maximum at about 300–800 ms under both modalities: in different cross-modal regions, the maximums of probability density functions occurred at 310–831 ms for the auditory modality, and the maximums occurred at 278–772 ms for the visual modality.

### 3.4. Classification Performance With Rest State

**Figure 11:**
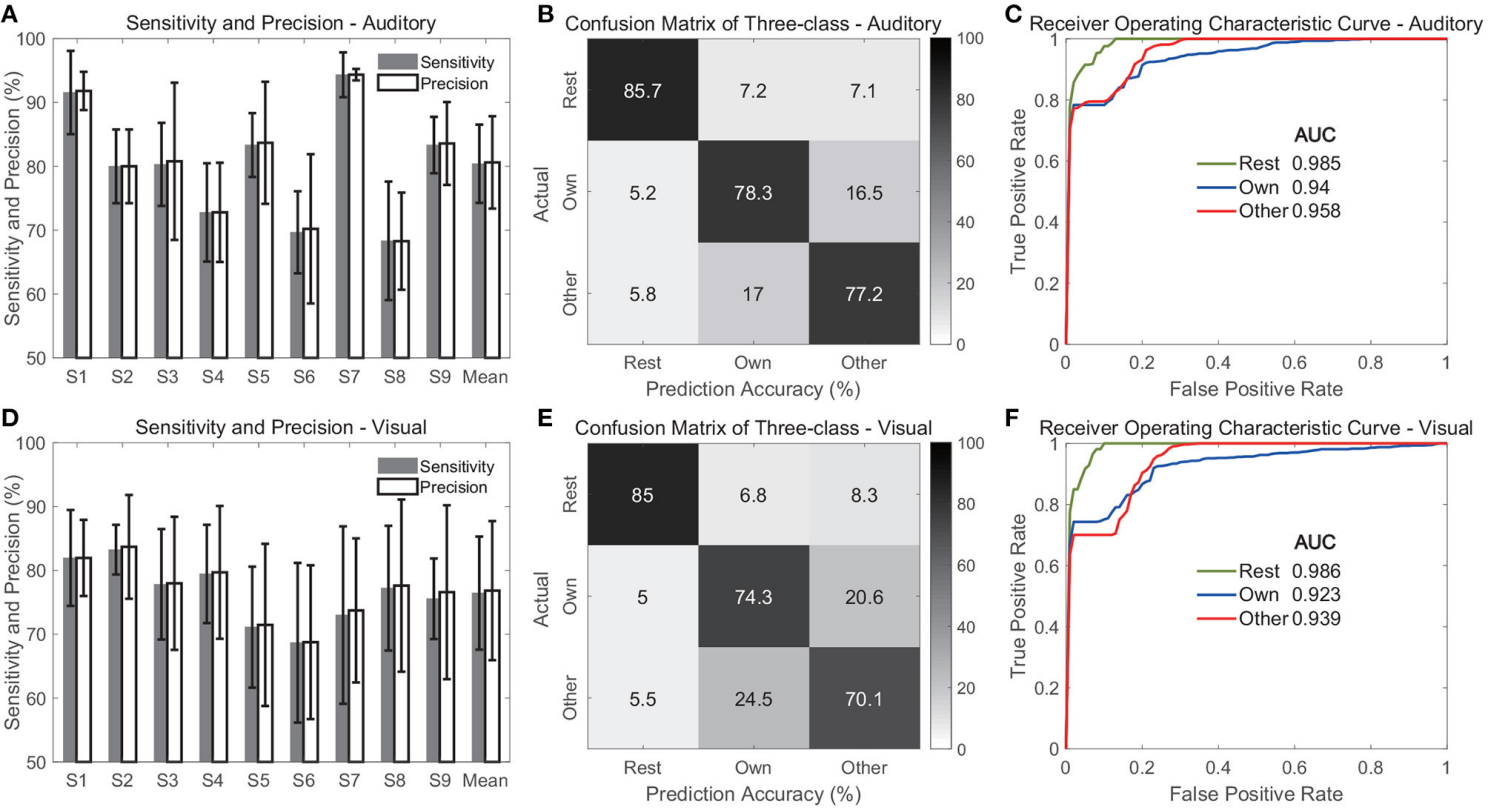
Classification performance for three classes including rest state. **(A–C)** Show evaluation of the auditory modality. **(D–F)** Show evaluation of the visual modality. Specifically, **(A,D)** show the sensitivity and precision. In these two sub-figures, the bars indicate the mean across three classes, and the error bars indicate the standard deviation across classes. **(B,E)** Show the average confusion matrices across subjects, and **(C,F)** show the average receiver operating characteristic curves of three classes across subjects.

Figure 11 shows the evaluation of the three-class classification performance when multi-region contacts and high-gamma band features were used. The average sensitivities across classes were 80.4 ± 6.1 and 76.4 ± 8.8% for the auditory and visual modalities, respectively (Figures 11A,D), which were slightly lower than the accuracies in the above two-class classification. Figures 11A,D also show the average precisions across classes, and the values were 80.6 ± 7.2 and 76.8 ± 10.9% in the auditory and visual modality, respectively. Further, the confusion matrices showed that the rest state achieved the highest average accuracy among the three classes, and the values were 85.7 ± 5.9 and 85 ± 3.7% in the auditory and visual modality, respectively (Figures 11B,E). However, the average accuracy for each name stimulus was much lower, with a range from 70.1 to 78.3% in both modalities. This finding indicated that it was relatively easy to discriminate the rest state from different stimuli states because the features in the rest state were different from features under name stimuli. Therefore, the ROC curve of the rest state showed the highest area under curve (AUC), and the values were 0.985 and 0.986 in the auditory and visual modalities (Figures 11C,F).

## 4. Discussion

### 4.1. Influencing Factors of Classification Performance

In this study, we demonstrated the feasibility of classifying the subject's name and the other name using SEEG from multiple brain regions. The accuracies were 84.5 ± 8.3% in the auditory modality and 79.9 ± 4.6% in the visual modality, where spatiotemporal features of high-gamma power achieved the best classification performance (Figure 4). When the rest state was added for the control scenario, the three-class classification accuracies did not show sharp declines because the high-gamma activity in the rest state was different from activations of name stimuli, which could be captured by the feature extraction approach relatively easily. The performance of high-gamma band was in accordance with the results reported in motor-task studies (Chestek et al., 2013; Bleichner et al., 2016). These excellent performances of the high-gamma band had potential physiological interpretations. The high-gamma band is considered to reflect the local neural population's activity directly around or underneath the recording electrode (Ray et al., 2008). Thus, the high-gamma activity indicates the excitability of the local region (Cardin et al., 2009; Parvizi and Kastner, 2018), and could be used as a reliable index to discriminate the task state from the rest state (Li et al., 2018a). On the other hand, accuracies computed using the beta and alpha bands could also exceed the significance levels (Figure 4). As we know, low-frequency oscillations in beta and alpha bands are considered carrier frequencies for communication between distant brain regions (Potes et al., 2014; Parvizi and Kastner, 2018). It was likely that the beta and alpha band provided a degree of Supplementary Material for the classification besides the high-gamma band, because beta and alpha activity may modulate the high-gamma response, especially in cognitive tasks (Parvizi and Kastner, 2018).

We noticed that classification accuracies varied across subjects (Figures 4A,B). This might be caused by individual differences in electrode implantation. The most typical example was subject S1, S7, and S9's auditory session (Figure 4A). These cases achieved high accuracies (93.4% on average) with a large number of electrode contacts (24 on average) in their STG and transverse temporal gyrus, covering parts of the primary auditory cortex. In contrast, subjects S2, S4, S5, and S6 had a comparatively small number of contacts in STG (11 on average), which might not capture enough information in the primary auditory cortex, and thus, might account for the inferior accuracies in the auditory session. The current result also emphasized the importance of accurately localizing the selective neural regions in further decoding study.

### 4.2. Significance of Cross-Modal Regions

In this work, we found some regions produced cross-modal responses, and these regions were more likely name-related (Tacikowski et al., 2013). The classification accuracies of these cross-modal regions exceeded the significance level (Figure 7 and Supplementary Figure 1). It has been known that the physical features of stimuli in the auditory and visual modality are processed in the primary auditory cortex and primary visual cortex, respectively, and these two functional areas locate differently (Brodmann, 2007). STG was a cross-modal region in the current study. Not surprisingly, STG showed high accuracy in subjects S1, S7, and S9's auditory sessions; this was possible because the auditory cortex in this region was sensitive to different phoneme stimuli (Mesgarani et al., 2014). Interestingly, as a part of the auditory cortex, STG also showed significant accuracy in subjects S1, S2, S4, and S9's visual session in the current study. Previous studies have shown that STG is a higher-order visual region involved in the analysis of biological stimuli and can be activated by observation of biological motion (Matthys et al., 2009), it also participates in visual search, spatial perception (Ellison et al., 2004; Gharabaghi et al., 2006), and speech perception via facial expressions (Reale et al., 2007). Moreover, brain responses in STG during social cognition were significantly reduced in patients with borderline personality disorder and ASD compared to controls (Dziobek et al., 2011; Mathersul et al., 2013). STG also showed greater activation to one's own name, self-related name, or familiar name than a stranger's name (Carmody and Lewis, 2006; Tacikowski et al., 2013). Therefore, parts of the STG might process cross-modal information and even the high-order cognitive process of self-related information. While auditory accuracies in STG were much higher than visual ones, the visual accuracies still exceeded the chance level significantly. One possible explanation was that parts of STG were involved in high-order cognition processing in both auditory and visual sessions. Especially, it also participated in low-order sound processing. Additionally, there might exist other possibilities. An fMRI study scanned both the auditory cortex and the visual cortex, and suggested that aurally presented names elicited more extensive and more reliable activations than visually presented names (Tacikowski et al., 2013). This phenomenon might be natural because people more often receive names by spoken versions in everyday life (Nowicka et al., 2016).

SMG was the most common cross-modal region in all subjects, suggesting its partial roles in self-related or name information processing. Similarly, studies have shown that self-other distinction in the emotional domain might be subserved by SMG (Sugiura et al., 2006; Silani et al., 2013), and even spared self-other distinction during empathy in ASD was relevant to an intact SMG network (Hoffmann et al., 2016).

As the second most common cross-modal region in this study, MTG showed greater activation to one's own name in fMRI studies (Carmody and Lewis, 2006), its activation was also displayed when the subject recognized characteristics that described himself/herself (Feng et al., 2018). Also, it was reported that MTG and SMG might play a role in word recognition, which mapped the physical structure of stimuli to the lexicon and accessed a lexical candidate (Stoeckel et al., 2009; Tacikowski et al., 2011). Therefore, it was probably that high-order roles of SMG and MTG in self-other distinction or word recognition led to response differences in both modalities.

Further, in a hypothetical case, if the classification accuracy of two names was mainly caused by physical differences between the stimuli, there would be a negative correlation between the cross-correlation value of stimuli and the classification accuracy. However, we did not find a correlation between the two measurements in these cross-modal regions under both modalities. This finding further reduced the possibility that the difference between responses in these cross-modal regions was caused by low-order physical stimuli. Therefore, it was more likely that the classification performance was associated with high-order cognition. Additionally, for both modalities, the analyses of response latency showed there was significant early activation for own name and other name, with an average latency in the range from 72 to 114 ms. These latencies were close to some ERP components including P100 (a positive component peaking approximately 100 ms after the stimulus onset) and N170 (a negative deflection reaching its maximum 170 ms after the stimulus onset), which reflect exogenous processes modulated by physical attributes of stimuli but not by cognitive processes (Coles and Rugg, 1995; Cygan et al., 2014). In contrast, the own-other difference had a longer latency of 354 ± 63 and 285 ± 59 ms in the auditory and visual modalities. These latencies were close to the P300 (a positive component peaking ~300 ms after the stimulus onset), which is considered to be an endogenous potential, reflecting decision making, stimulus evaluation, and recognition (Coles and Rugg, 1995; Smigielski et al., 2020). Therefore, the current cross-modal regions might process low-order physical information of the stimuli at about 100 ms after the stimuli onset, and then the meaning of names might be processed at about 300 ms after the stimuli onset. The significant response difference after about 300 ms mainly contributed to the classification. Analyses for the generation times of features in *V*_20_ suggested that the two names could be distinguished best at about 300–800 ms after the stimulus onset. This time range was close to the results of scalp-EEG studies, which suggested that ERPs between 350 and 850 ms after the stimulus onset were the most distinguishable for different names (Tacikowski and Nowicka, 2010). Especially, the current study located precise cross-modal regions that generated the response difference of cognitive contents, which couldn't be achieved by scalp-EEG measurements.

All subjects presented at least seven cross-modal regions. It was feasible to decode cognitive contents using these regions (Figure 7 and Supplementary Figure 1), where STG, MTG, middle frontal gyrus, insula, fusiform gyrus, and SMG are discussed in previous name-related researches (Carmody and Lewis, 2006; Sugiura et al., 2006; Tacikowski et al., 2011, 2013; Qin et al., 2012). In the macroscopic view, these critical cross-modal name-related regions spanned the frontal, temporal, and parietal lobe, indicating that the processing of name-recognition involves complex distributed networks (Qin and Northoff, 2011; Tacikowski et al., 2011).

### 4.3. Implications

Awareness and cognition assessment by direct observation of the responses might be influenced by inherent variabilities among individuals. For ease of comparison, the classification accuracy of responses to different stimuli has seemed like an alternative evaluation index to compensate for the above shortcoming (King et al., 2013). A study has detected potential awareness in patients with DOC via a hybrid BCI (Pan et al., 2014), owing to the promising classification accuracy of the patient's facial photo and an unknown facial photo. Studies have also shown that name-response and facial photo-response have a variety of similar components and trends (Tacikowski and Nowicka, 2010; Pawel et al., 2011; Cygan et al., 2014). Therefore, the classification accuracy of name-responses is another potential index for conscious awareness detection and cognitive function evaluation. In a scalp-EEG study, aurally presented own name and other name could be distinguished using P300 with accuracies of 80.9 and 74.5%, when the length of names was three Chinese characters and two Chinese characters, respectively (Yang et al., 2020). The accuracy using scalp-EEG was slightly lower than the auditory accuracy using high-gamma band (84.5%) in the current study. Besides, our name classification framework could serve for the decoding process of a bidirectional cognitive BCI system. Depth electrodes also provided a way for deep brain electrical stimulation in the meantime, where cross-modal regions were potential target regions of stimulation for patients with disorders of cognition and consciousness (Lemaire et al., 2014; Sankar et al., 2014). Gradual improvements in techniques of name decoding would benefit patients with ASD and DOC eventually.

### 4.4. Limitations and Future Work

Even though we have demonstrated the possibility of decoding names using SEEG signals, there were several limitations in this work. We only presented the subject's name and a stranger's name to patients to limit their psychological burden. In future work, we would present more diverse names to a larger number of subjects to further exclude the possibility of physical features of stimuli. Also, the names would be subdivided into different levels of familiarity and emotional content to generate more comprehensive evaluations. Additionally, connectivity networks involving in name processing would be further investigated using distributed SEEG.

## 5. Conclusion

We provided a decoding framework for name processing using SEEG. The results of the current work indicated the feasibility of decoding names from both multiple brain regions and a certain crucial region. Cross-modal significant classification accuracies in a single region might locate subsets of the name-recognition network. Abundant spatiotemporal information of SEEG provides new insights into the cognitive processes and would encourage further clinical applications.

## Data Availability Statement

The original contributions presented in the study are included in the article/Supplementary Material, further inquiries can be directed to the corresponding author/s.

## Ethics Statement

The studies involving human participants were reviewed and approved by Ethics Committee of Huashan Hospital. Written informed consent to participate in this study was provided by the participant or his/her legal guardian/next of kin.

## Author Contributions

HY, ZF, and LC: conceptualization. HY and GC: methodology. HY and GL: software. HY and ZF: formal analysis, data curation, writing-original draft, and visualization. HY, ZF, ZW, XS, XZ, and LC: investigation. JH, XS, XZ, and LC: resources. GL and GC: writing-review and editing. XZ, LC, and JH: supervision. XZ, LC, and XS: project administration and funding acquisition. All authors read and approved the final version of the manuscript.

## Conflict of Interest

The authors declare that the research was conducted in the absence of any commercial or financial relationships that could be construed as a potential conflict of interest.
