# Supplementary material for: Self-Related Stimuli Decoding With Auditory and Visual Modalities Using Stereo-Electroencephalography
Source: Front Neurosci. 2021 May 4;15:653965. doi: 10.3389/fnins.2021.653965 (PMC8129191; doi:10.3389/fnins.2021.653965)
Supplement: Supplementary file 1 [file Presentation_1.pdf]

Supplementary Material

1 SUPPLEMENTARY TEXTS

Figure S1 shows auditory and visual classification accuracies in all cross-modal regions for all subjects.

For comparison between features, we investigated the classification performance of another summative measure, the line-length feature of broad-band signal. The contacts from multiple regions were used to generate effective features for the three-class classification. For the 1000-ms rest state and stimuli state, the whole high-gamma time series was divided into ten 100-ms bins, and then we calculated the line-length in each bin to generate the feature. The dimensional reduction procedure was the same as that in section 2.8. The classification performance was further assessed in terms of sensitivity and precision, confusion matrix, and ROC curve.

Figure S2 shows three-class classification performance using line-length features. The average sensitivities across classes were 77.6 $\pm$ 7.8% and 72.8 $\pm$ 7.6% for the auditory and visual modalities, respectively (Figure S2A and D), which were slightly lower than the sensitivities in the three-class classification using average high-gamma power in 10-ms bins. The average precisions across classes were 78 $\pm$ 10.3% and 73.3 $\pm$ 11.3% for the auditory and visual modalities, respectively. Further, the confusion matrices showed that the rest state achieved the average accuracies of 84% and 80.9% in the auditory and visual modality, respectively (Figure S2B and E), while the average accuracy for each name stimulus was much lower, with a range from 67.4% to 75.4% in both modalities. The ROC curve of the rest state showed the highest AUC, and the values were 0.985 and 0.981 in the auditory and visual modalities (Figure S2C and F). These values were still lower than ones using average power features in 10-ms bins. In conclusion, the line-length feature in 100-ms bins got slightly lower performance than the average power feature in 10-ms bins.

2 SUPPLEMENTARY FIGURES

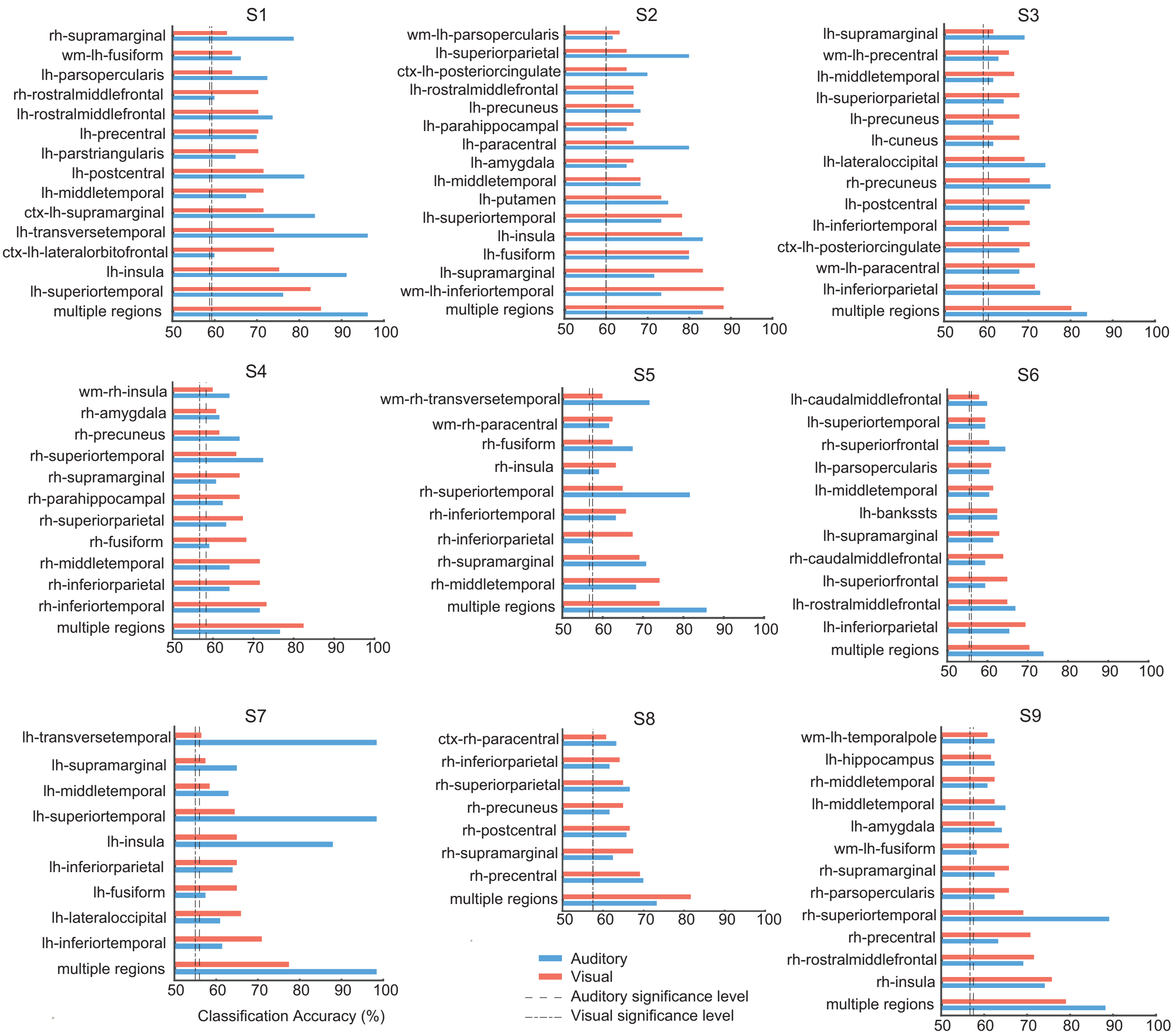

Figure S1. Classification performance of all cross-modal regions for all subjects. For each subject, the classification was performed within each single brain region. Only regions that achieved significant accuracies in both modalities are shown here. As a comparison, the significance levels of classification based on multiple regions are shown, where short dashed line and long dashed line indicate the auditory and visual session, respectively. Abbreviations are used to point out brain regions where contacts are located: lh for left hemisphere, rh for right hemisphere, ctx for cortex, wm for white matter, respectively.

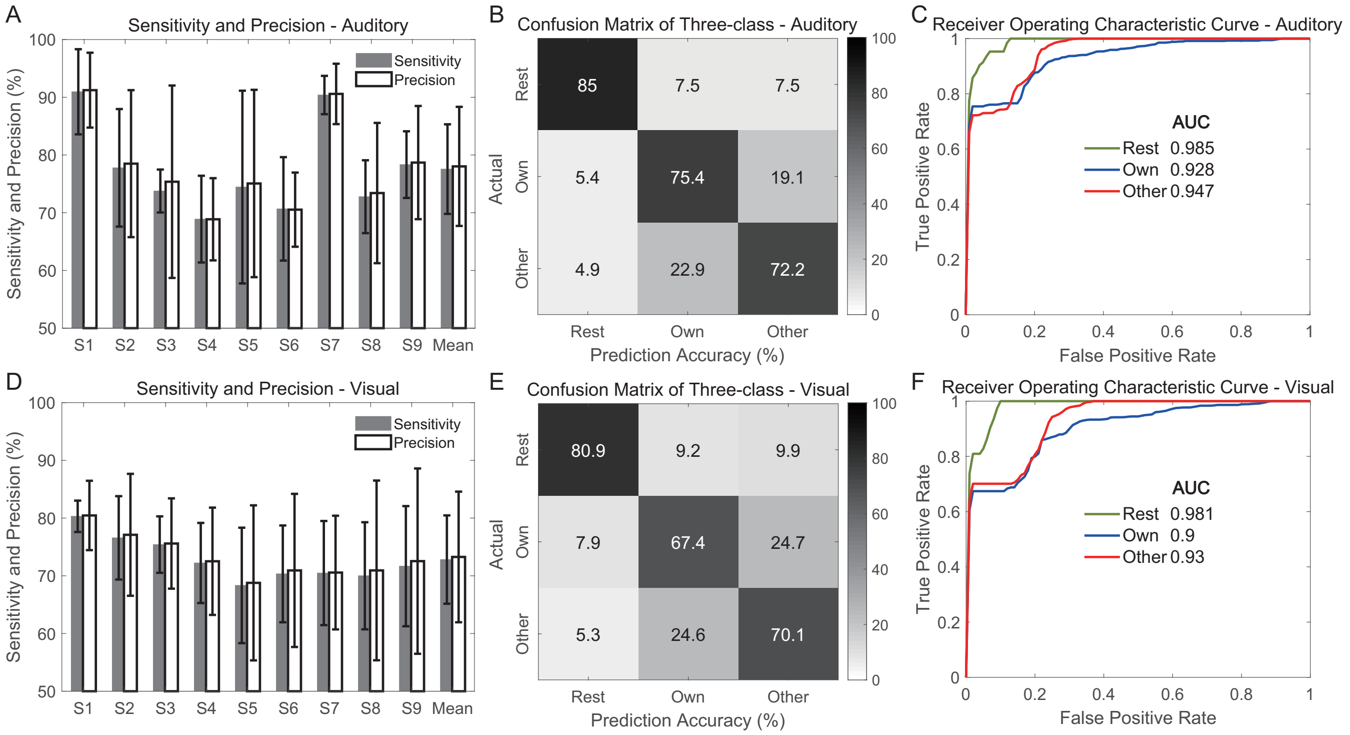

Figure S2. Classification performance for three classes with rest state using line-length features. **(A)**, **(B)**, and **(C)** show evaluation of the auditory modality. **(D)**, **(E)**, and **(F)** show evaluation of the visual modality. Specifically, **(A)** and **(D)** show the sensitivity and precision. In these two sub-figures, the bars indicate the mean across three classes, and the error bars indicate the standard deviation across classes. **(B)** and **(E)** show the average confusion matrices across subjects, and **(C)** and **(F)** show the average receiver operating characteristic curves of three classes across subjects.
